# AMG 837: A Novel GPR40/FFA1 Agonist that Enhances Insulin Secretion and Lowers Glucose Levels in Rodents

**DOI:** 10.1371/journal.pone.0027270

**Published:** 2011-11-08

**Authors:** Daniel C.-H. Lin, Jane Zhang, Run Zhuang, Frank Li, Kathy Nguyen, Michael Chen, Thanhvien Tran, Edwin Lopez, Jenny Ying Lin Lu, Xiaoyan Nina Li, Liang Tang, George R. Tonn, Gayathri Swaminath, Jeff D. Reagan, Jin-Long Chen, Hui Tian, Yi-Jyun Lin, Jonathan B. Houze, Jian Luo

**Affiliations:** 1 Metabolic Disorders, Amgen Inc., South San Francisco, California, United States of America; 2 Chemistry Research and Discovery, Amgen Inc., South San Francisco, California, United States America; 3 Pharmacokinetics and Drug Metabolism, Amgen Inc., South San Francisco, California, United States America; University of Tor Vergata, Italy

## Abstract

Agonists of GPR40 (FFA1) have been proposed as a means to treat type 2 diabetes. Through lead optimization of a high throughput screening hit, we have identified a novel GPR40 agonist called AMG 837. The objective of these studies was to understand the preclinical pharmacological properties of AMG 837. The activity of AMG 837 on GPR40 was characterized through GTPγS binding, inositol phosphate accumulation and Ca^2+^ flux assays. Activity of AMG 837 on insulin release was assessed on isolated primary mouse islets. To determine the anti-diabetic activity of AMG 837 *in vivo*, we tested AMG 837 using a glucose tolerance test in normal Sprague-Dawley rats and obese Zucker fatty rats. AMG 837 was a potent partial agonist in the calcium flux assay on the GPR40 receptor and potentiated glucose stimulated insulin secretion *in vitro* and *in vivo*. Acute administration of AMG 837 lowered glucose excursions and increased glucose stimulated insulin secretion during glucose tolerance tests in both normal and Zucker fatty rats. The improvement in glucose excursions persisted following daily dosing of AMG 837 for 21-days in Zucker fatty rats. Preclinical studies demonstrated that AMG 837 was a potent GPR40 partial agonist which lowered post-prandial glucose levels. These studies support the potential utility of AMG 837 for the treatment of type 2 diabetes.

## Introduction

GPR40 (also known as FFA1 and FFAR1) is a free fatty acid-activated G protein-coupled receptor that is found on the surface of pancreatic β-cells, gastrointestinal enteroendocrine cells, immune cells and parts of the brain. Long chain saturated and unsaturated fatty acids stimulate GPR40, and evidence points to GPR40 being a mechanistic link to the well-known effects of fatty acids to acutely stimulate insulin and incretin secretion [1], [2], [3]. The effect of fatty acids on insulin and incretin (glucagon-like peptide-1 (GLP-1) and glucose-dependent insulinotropic peptide (GIP)) secretion is blunted or eliminated in mice lacking GPR40 [4]. GPR40 knockout mice also show impaired glucose and arginine induced insulin secretion *in vivo* [5]. Based on these studies, targeting GPR40 with synthetic agonists may represent a novel pathway in the treatment of type 2 diabetes. Because activity of GPR40 agonists on islet β-cells is glucose dependent, it is believed that GPR40 may offer advantages to commonly used sulfonylurea drugs which act independently of ambient glucose levels, resulting in hypoglycemia in some patients [6].

The molecular mechanisms of GPR40-mediated signal transduction have been best studied in pancreatic beta cell lines (*eg* MIN-6 and INS-1) and primary pancreatic β-cells. GPR40 couples to the G_αq_ class of G-proteins, leading to the formation of inositol phosphate and increases in intracellular calcium. GPR40 enhancement of glucose-stimulated insulin secretion requires extracellular calcium [1]. Using isolated rat pancreatic β-cells, Fujiwara *et al* demonstrated that GPR40 mediated increases in intracellular calcium were glucose-dependent, but independent of the endoplasmic reticulum Ca^2+^ pump [7]. Increases in intracellular Ca^2+^ and insulin release were mediated through activity of phospholipase C and an L-type Ca^2+^ channel. Further, stimulation of GPR40 in pancreatic β-cells led to attenuation of a voltage-gated potassium channel in a protein kinase A dependent pathway [8].

Both small molecule agonists and antagonists of GPR40 have been described [9], [10], [11], [12], [13], [14]. Consistent with its activity in isolated islets, GPR40 agonists *in vivo* improved post-prandial glucose tolerance in rodents following acute administration. TAK-875 [15], [16] and AMG 837, described in this paper, represent the first GPR40 agonists that have entered clinical trials.

GPR40 is detected in human islets samples from multiple donors [17], [18] and clinical studies with GPR40 agonists will aid in elucidating the function of GPR40 in humans. Several single nucleotide polymorphisms (SNP) in the human GPR40 gene have been described which may shed light on the function of GPR40 in humans. The most common human SNP has been identified in the coding region of GPR40 and results in an arginine at position 211 in place of a histidine. Using homeostasis modeling of β-cell function, Ogawa *et al* reported that the His211Arg polymorphism may contribute to a variation in insulin secretory capacity [19]. However, a study by Hamid *et al* analyzing healthy and type 2 diabetic Danish subjects concluded that there was no association of the SNP at codon 211 and type 2 diabetes or insulin release [20]. Furthermore, in cell-based functional assays the Arg211 and His211 variants responded identically to fatty acids [20]. Another variant in the GPR40 gene, Gly180Ser, displayed reduced response *in vitro* to fatty acids and also diminished insulin secretory capacity in human carriers, suggesting an important role of GPR40 in insulin secretion in humans [21]. Finally, a recent report of a mouse transgenic model containing human GPR40 under control of the insulin promoter showed improved glucose tolerance and islet physiology [22]. A potent, specific GPR40 agonist used in a clinical setting will clarify the biology of GPR40 in humans.

In this report, we describe the preclinical pharmacological characterization of a novel synthetic GPR40 agonist, AMG 837. AMG 837 stimulates glucose dependent insulin secretion in rodent islets in a GPR40-dependent manner. *In vivo*, AMG 837 improved glucose tolerance through stimulation of insulin secretion in both normal and Zucker fatty rats. The efficacy persisted after daily dosing of AMG 837 for 21-days in Zucker fatty rats. These data support the further development of AMG 837 in the clinic.

## Results

### *In vitro* characterization of AMG 837

**Figure 1:**
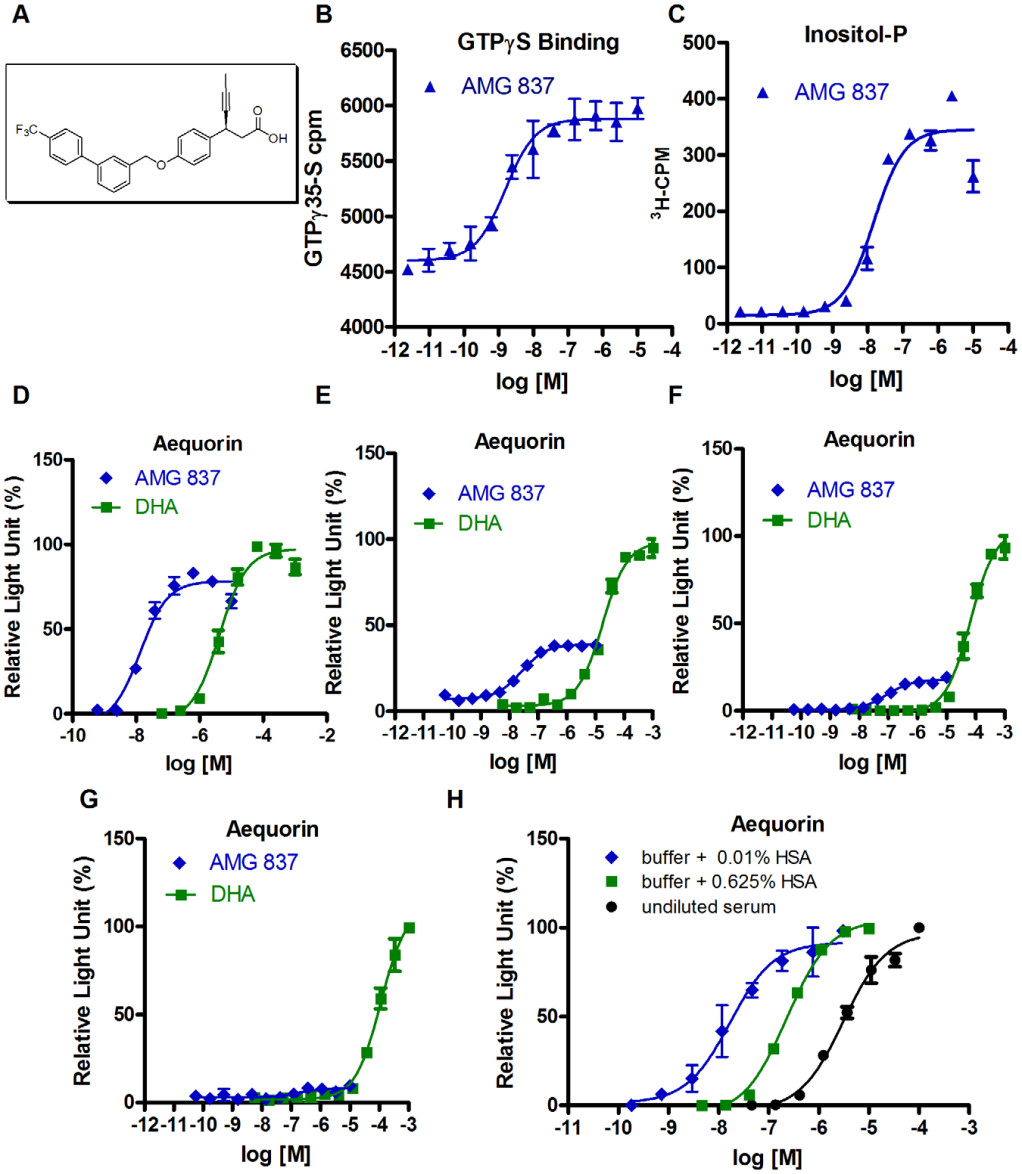
*In vitro* characterization of AMG 837. (A) The chemical structure of AMG 837 is shown. (B–D) The activity of AMG 837 in various GPCR assays was assessed as described in Materials and Methods. Dose response relationships of AMG 837 in GTPγS binding (B), inositol phosphate accumulation (C) and aequorin Ca^2+^ flux assays (D) in cell lines overexpressing GPR40/FFA1 were determined. (D–G) In order to compare the activity of AMG 837 to fatty acids, plasmid titration experiments where either 5000 ng (D), 500 ng (E), 50 ng (F) or 5 ng (G) of GPR40 expression plasmid was co-transfected with aequorin expression plasmids into CHO cells. Activity of AMG 837 (blue diamond) was compared to the naturally occurring GPR40/FFA1 ligand docosahexaenoic acid (DHA, green square) in aequorin Ca^2+^ flux. (H) The activity of AMG 837 in the aequorin Ca^2+^ flux assays in the presence of 0.01% (v/v) purified human serum albumin (HSA, blue diamond), 0.625% (w/v) HSA (green square) or human serum (100% v/v, black circle) was determined.

A high throughput screen for GPR40 agonists resulted in the identification of a lead series of β-substituted phenylpropanoic acids that was further optimized to obtain AMG 837. AMG 837 features an alkyne at the β-position relative to the carboxylic acid and a substituted biaryl group remote to the acid that increases potency on GPR40 relative to the lead series (Houze JB *et al*, in preparation). The structure of AMG 837 is shown in figure 1A.

Activity of AMG 837 on GPR40 was characterized in a variety of biochemical and cell-based assay using cell lines that stably or transiently expressed GPR40. Because GPR40 is coupled to the G_αq_ class of G-proteins, we measured agonist-stimulated [^35^S]-GTPγ binding using an antibody capture method [23]. Cell membranes were prepared from an A9 derived cell line stably overexpressing human GPR40 (A9_GPR40). Incubation of GPR40 containing cell membranes with AMG 837 increased [^35^S]-GTPγ binding with an EC_50_ of 1.5±0.1 nM (n = 2, figure 1B). This is the first report of a [^35^S]-GTPγ binding assay for GPR40 using antibody capture to G_αq_ and further confirms that GPR40 couples to G_αq_.

Activity of AMG 837 on GPR40 was further explored in cell-based functional assays for the second messengers inositol phosphate and intracellular Ca^2+^. These assays were done in the presence of a low concentration of HSA (human serum albumin; 0.01% w/v) in order to minimize the effect of binding of AMG 837 to albumin (further described below). AMG 837 stimulated inositol phosphate accumulation with an EC_50_ of 7.8±1.2 nM (n = 52) in the A9_GPR40 cell line (figure 1C) that is consistent with the potency observed for GTPγS binding. Changes in intracellular calcium were measured using the Ca^2+^ sensitive bioluminescent aequorin reporter. In CHO cells transiently transfected with human GPR40 and aequorin, AMG 837 stimulated Ca^2+^ flux with an EC_50_ of 13.5±0.8 nM (n = 153) on the human GPR40 receptor (table 1, figure 1D). AMG 837 did not have activity on the related receptors GPR41 (FFA2), GPR43 (FFA3) or GPR120 at concentrations up to 10 µM, indicating that the activity was specific to GPR40 (table 1). The EC_50_ of AMG 837 in the aequorin assay was 22.6±1.8 nM (n = 37), 31.7±1.8 nM (n = 53), 71.3±5.8 (n = 7) and 30.6±4.3 nM (n = 25) on mouse, rat, dog and rhesus monkey GPR40, respectively (table 1). These results, coupled with the favorable pharmacokinetic profile of AMG 837 (Houze JB *et al*, in preparation), indicate that the pharmacology of AMG 837 could be studied in common rodent and non-human primate preclinical species.

**Table 1:** Aequorin Ca^2+^ Flux Activity (EC_50_, nM) of AMG 837 on Various Receptors.

species	Human	Mouse	Rat	Dog	Monkey	Human	Human	Human
**receptor**	GPR40	GPR40	GPR40	GPR40	GPR40	GPR41	GPR43	GPR120
**EC_50_, nM**	13.5±0.8	22.6±1.8	31.7±1.8	71.3±5.8	30.6±4.3	>10,000	>10,000	>10,000

CHO cells were co-transfected with expression plasmids of a given receptor along with the Ca^2+^ sensitive bioluminescent reporter aequorin, as described in Materials and Methods. Response to AMG 837 was measured with a luminometer and the EC_50_ (nM) ± SEM was determined.

In order to explore whether AMG 837 was a full or partial agonist of GPR40, we compared the activity of AMG 837 to that of the naturally occurring fatty acid ligand, docosahexaenoic acid (DHA), in plasmid titration experiments. In our standard aequorin assay, 5 µg of GPR40 expression plasmid (GPR40 under the control of the CMV promoter) are used to transfect ∼10–12 million CHO cells. Under these conditions, AMG 837 behaved as a partial agonist on the GPR40 receptor when compared to DHA, with a maximal activity 85% that of DHA (figure 1D). Because the relative activity of partial and full agonists is dependent on receptor expression levels, we reduced the amount of GPR40 plasmid that was transfected by ten-fold increments. The total amount of plasmid transfected was kept constant by adding an appropriate amount of empty vector DNA. Under conditions in which 0.5, 0.05, and 0.005 µg of GPR40 expression plasmid were transfected to an equivalent number of cells in parallel, the maximum agonist response of AMG 837 was 40%, 20%, and 10% as compared to the maximal effect of DHA, respectively (figure 1E,F,G). These results confirm that AMG 837 is a partial agonist on the GPR40 receptor in this assay format.

AMG 837 is 98.7% bound when incubated with human plasma, indicating extensive binding to plasma proteins. Consistent with this, we found that the EC_50_ of AMG 837 in the GPR40 aequorin assay was ∼180-fold less potent when tested in the presence of human serum (100% v/v, EC_50_ = 2,140±310 nM (n = 7)) compared to the assay in 0.01% HSA (figure 1H). Because human serum albumin is well known to bind fatty acids and xenobiotics, we tested the effect of delipidated human serum albumin on AMG 837 activity. The activity of AMG 837 was reduced approximately 16-fold (EC_50_ of 210±12 nM, n = 42) in the presence of 0.625% delipidated HSA compared to the assay in the presence of 0.01% HSA (figure 1H), indicating that AMG 837 likely binds to albumin.

### Potentiation of Insulin Secretion by AMG 837 in Isolated Islets

**Figure 2:**
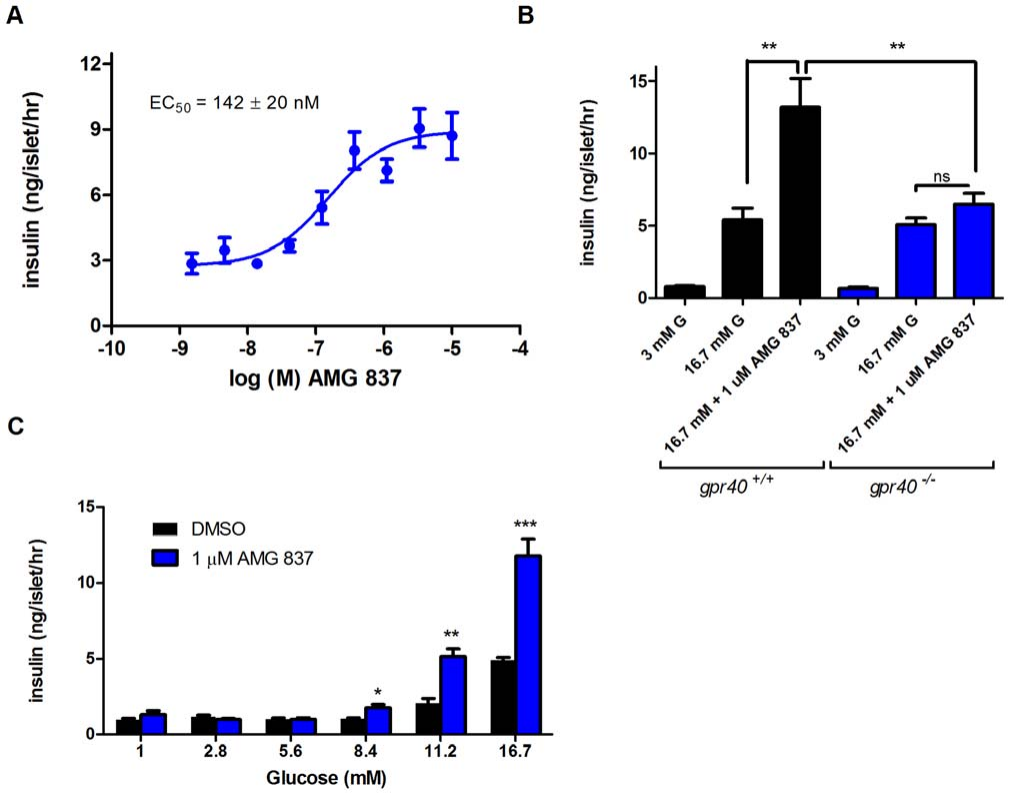
AMG 837 Potentiates Insulin Secretion from Islets. Islets were isolated from mice and the activity of AMG 837 on insulin secretion was determined. (A) The dose response relationship of AMG 837 and insulin secretion on mouse islets at 16.7 mM glucose was evaluated. (B) In order to determine whether the activity of AMG 837 was GPR40/FFA1 dependent, islets were isolated from GPR40 null mice (*gpr40^−/−^*). AMG 837 potentiated glucose stimulated insulin secretion from wild type islets (black bar), but not *gpr40^−/−^* islets (blue bar). (C) Glucose dependence of AMG 837 on glucose stimulated insulin secretion was determined by incubating islets in buffer containing either 0.1% DMSO (black bar) or 1 µM AMG 837 in 0.1% DMSO (blue bar) in the presence of increasing concentrations of glucose. Statistical significance is denoted by * (p<0.5), ** (p<0.01) and *** (p<0.001) as determined by one-way or two-way ANOVA.

GPR40 is expressed predominantly in the β-cells of the pancreatic islet and activation of GPR40 improves glucose-stimulated insulin secretion (GSIS). We examined the activity of AMG 837 on isolated islets in order to understand the effect of the compound on a relevant primary cell type. On islets isolated from mice, AMG 837 stimulated insulin secretion with an EC_50_ of 142±20 nM (n = 3, figure 2A). The activity of AMG 837 was eliminated in islets isolated from GPR40 knockout mice (figure 2B), indicating that the activity of AMG 837 was indeed specific to GPR40.

GPR40 agonists have been reported to increase insulin secretion in a glucose- dependent manner, and similarly we found that the activity of AMG 837 was glucose dependent. Activation of GPR40 by AMG 837 did not result in potentiation of glucose stimulated insulin secretion (GSIS) at glucose concentrations ≤5.6 mM (figure 2C). At higher glucose concentrations (≥8.3 mM), AMG 837 increased GSIS. This suggests that the risk of hypoglycemia with AMG 837 may be lower compared with that of insulin secretagogues such as sulfonylureas that stimulate insulin secretion regardless of ambient glucose levels [6].

### AMG 837 Stimulates Insulin Secretion and Lowers Postprandial Glucose Levels in Normal Rodents

We next tested the ability of AMG 837 to improve glucose tolerance and stimulate insulin secretion in Sprague-Dawley rats. Sprague-Dawley rats were chosen since they are euglycemic, allowing AMG 837 to be tested at normal glucose levels and during the challenged state following a glucose bolus. AMG 837 displays excellent pharmacokinetic properties in multiple species (Houze JB *et al*, in preparation). The pharmacokinetic profile following a single 0.5 mg/kg oral dose in rats displayed excellent oral bioavailability (%F = 84) and a total plasma C_max_ of 1.4 µM. AMG 837 was dosed by oral gavage at 0.03 mg/kg, 0.1 mg/kg and 0.3 mg/kg 30 minutes prior to an intraperitoneal glucose challenge. Glucose and insulin levels were determined before and after administration of glucose.

**Figure 3:**
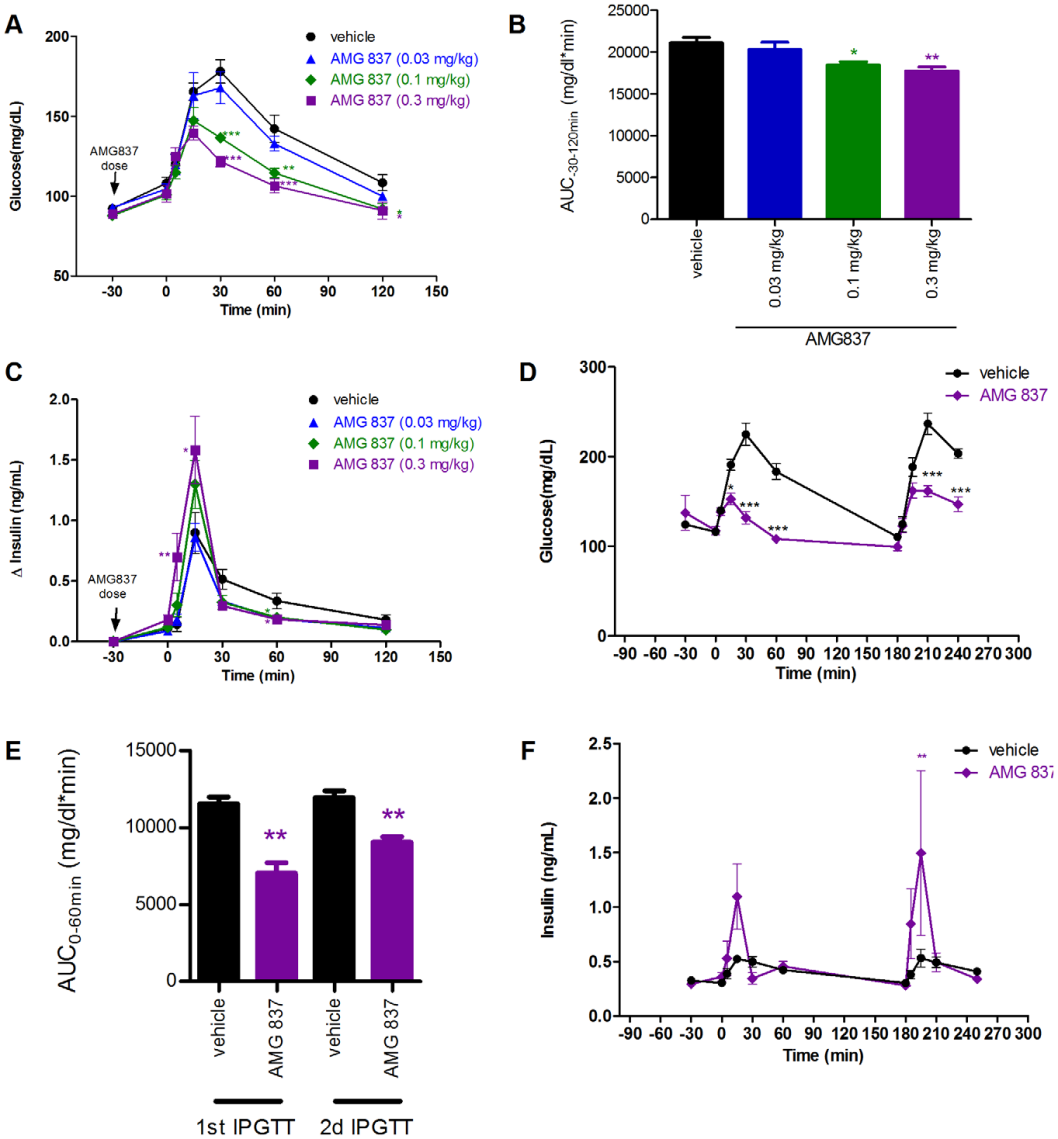
Improvement in glucose tolerance and potentiation of insulin secretion in Sprague-Dawley rats treated with AMG 837. 8-week old Sprague-Dawley rats were treated with a single bolus of AMG 837 (at 0.03, 0.1 and 0.3 mg/kg, n = 6/group) by oral gavage 30-minutes prior to an intraperitoneal glucose challenge at t = 0 minutes. (A) Blood glucose measurements were taken during prior to and following glucose challenge. Black circle = vehicle, blue triangle = 0.03 mg/kg AMG 837, green diamond = 0.1 mg/kg AMG 837 and purple square = 0.3 mg/kg AMG 837 (B) The glucose AUC (from −30 to 120 minutes) during the course of the experiments were calculated. (C) Plasma insulin levels were measured using ELISA. Black circle = vehicle, blue triangle = 0.03 mg/kg AMG 837, green diamond = 0.1 mg/kg AMG 837 and purple square = 0.3 mg/kg AMG 837 (D–D) Two successive glucose challenges were conducted in Sprague-Dawley rats following a single oral dose of vehicle (n = 4, black circle) or AMG 837 at 0.3 mg/kg (n = 4, purple diamond). AMG 837 was dosed at −30 minutes, and glucose was administered by *ip* injection at 0 and 180 minutes. Blood glucose (D), calculated glucose AUC (from 0–60 minutes following glucose challenge (E, black bars = vehicle, purple bars = 0.3 mg/kg AMG 837) and plasma insulin (F) were determined. Statistical significance is denoted by * (p<0.5), ** (p<0.01) and *** (p<0.001) as determined by one-way or two-way ANOVA.

AMG 837 administration did not have any effect on glucose levels prior to the glucose tolerance test (30 minutes following AMG 837 administration). Following administration of glucose, plasma glucose levels were suppressed in an AMG 837 dose-dependent manner (figure 3A). At the low, mid and high dose, glucose AUC improved 3.9%, 14.5% (p<0.05) and 18.8% (p<0.01) compared to that of vehicle treated animals, respectively (figure 3B). The half-maximal dose of AMG 837 to lower post-prandial glucose in rats was approximately 0.05 mg/kg.

The improvement in post-prandial glucose was a result of an increase in glucose-stimulated insulin secretion. In animals treated with AMG 837, there was a dose-dependent increase of plasma insulin levels following the glucose challenge (figure 3C). The increase of plasma insulin levels was rapid and of short duration, most evident at 5 and 15 minutes following glucose administration. Taken together, these results indicate that the activity of AMG 837 was dependent on glucose *in vivo*.

We further tested whether a single dose of AMG 837 could improve post-prandial glucose following consecutive glucose challenges. A single dose (0.3 mg/kg) of AMG 837 was administered to Sprague-Dawley rats followed by two intraperitoneal glucose challenges 3 hours apart. AMG 837 improved blood glucose levels during both glucose challenges (p<0.01, figure 3D, E). As observed in the single glucose challenge, peak insulin secretion during each glucose challenge increased soon after the glucose administration (figure 3F). These results indicate that pharmacological effect of a single dose of AMG 837 on pancreatic β-cells persists over the course of several hours.

### Efficacy of AMG 837 in Zucker Fatty Rats Following Once Daily Dosing for 21-days

**Figure 4:**
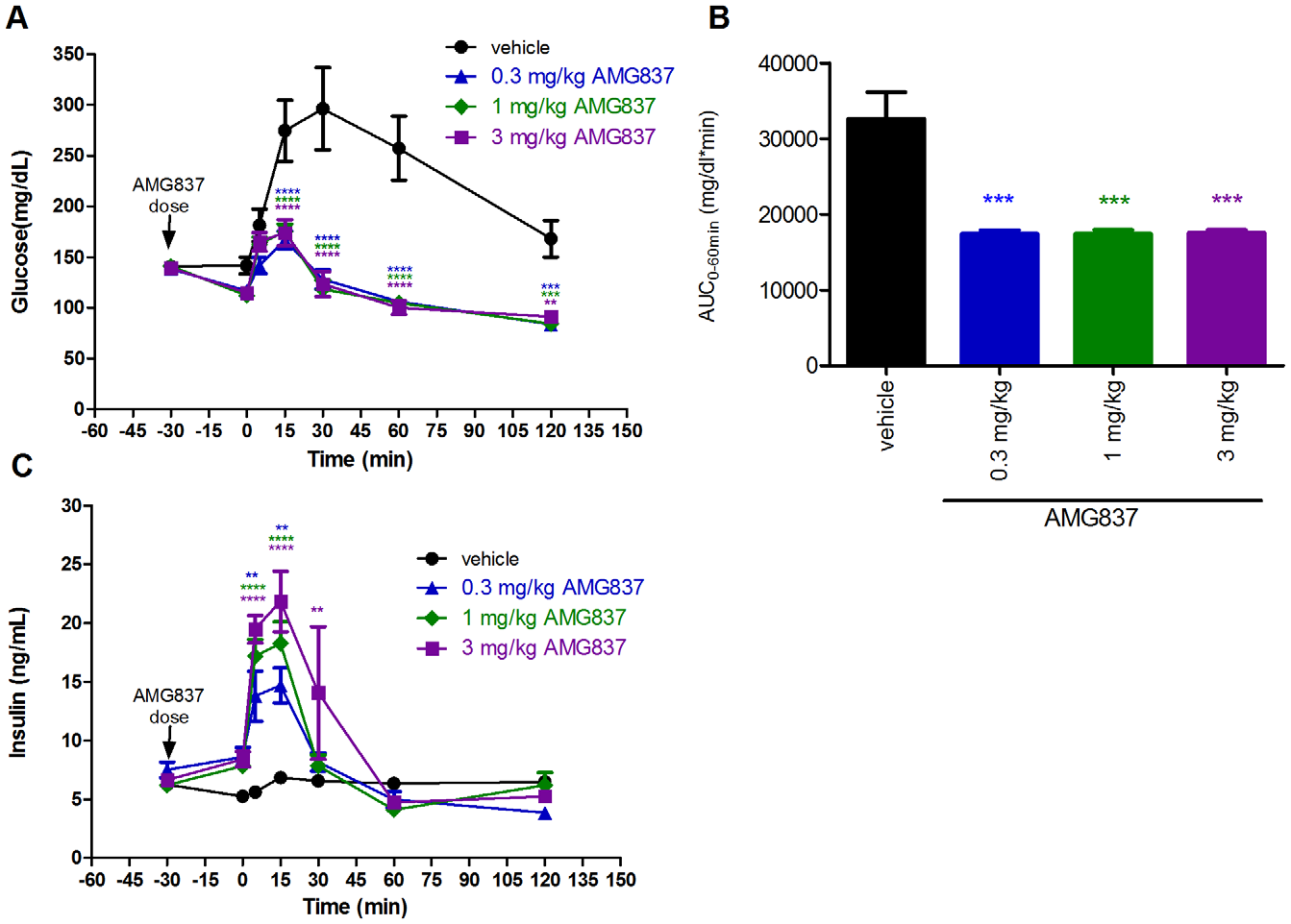
Efficacy of AMG 837 in Zucker fatty (*fa/fa*) rats following a single dose. 8-week old Zucker fatty rats were administered a single bolus of AMG 837 (at 0.3, 1 and 3 mg/kg, n = 6/group) by oral gavage 30-minutes prior to an intraperitoneal glucose challenge at t = 0 minutes. (A) Blood glucose during the IPGTT (black circle = vehicle, blue triangle = 0.3 mg/kg AMG 837, green diamond = 1 mg/kg AMG 837 and purple square = 3 mg/kg AMG 837) (B) Glucose AUC (from −30 to 120 minutes) during the IPGTT. (C) Plasma insulin levels during the IPGTT (black circle = vehicle, blue triangle = 0.3 mg/kg AMG 837, green diamond = 1 mg/kg AMG 837 and purple square = 3 mg/kg AMG 837). Statistical significance compared to vehicle treated animals is denoted by * (p<0.5), ** (p<0.01), *** (p<0.001) and **** (p<0.001) as determined by one-way or two-way ANOVA and colors match the corresponding groups in the figure legend.

We next tested the effect of AMG 837 in the insulin resistant Zucker fatty (*fa/fa*) rat following single and multiple doses of AMG 837. The Zucker fatty rat model was studied since it displays impaired glucose tolerance, hyperinsulinemia and mild hyperglycemia [24], [25]. AMG 837 was first tested in single doses of 0.3, 1 and 3 mg/kg prior to an IPGTT. In contrast to that observed in normal Sprague-Dawley rats, glucose levels 30 minutes following the AMG 837 dose trended lower and insulin levels trended higher, although neither parameter reached statistical significance (figure 4A,C). Because the activity of AMG 837 on GPR40 is glucose dependent, the higher basal glucose levels in insulin resistant Zucker fatty rats compared to that in Sprague-Dawley rats may be sufficient to trigger a response. Following the glucose challenge, glucose levels were lower at all doses of AMG 837 and the glucose excursion curves largely overlapped (figure 4A). The glucose AUC for all doses decreased ∼46% (p<0.001, figure 4B). As observed in Sprague-Dawley rats, plasma insulin levels spiked most prominently 5 and 15 minutes post glucose challenge (figure 4C).

**Figure 5:**
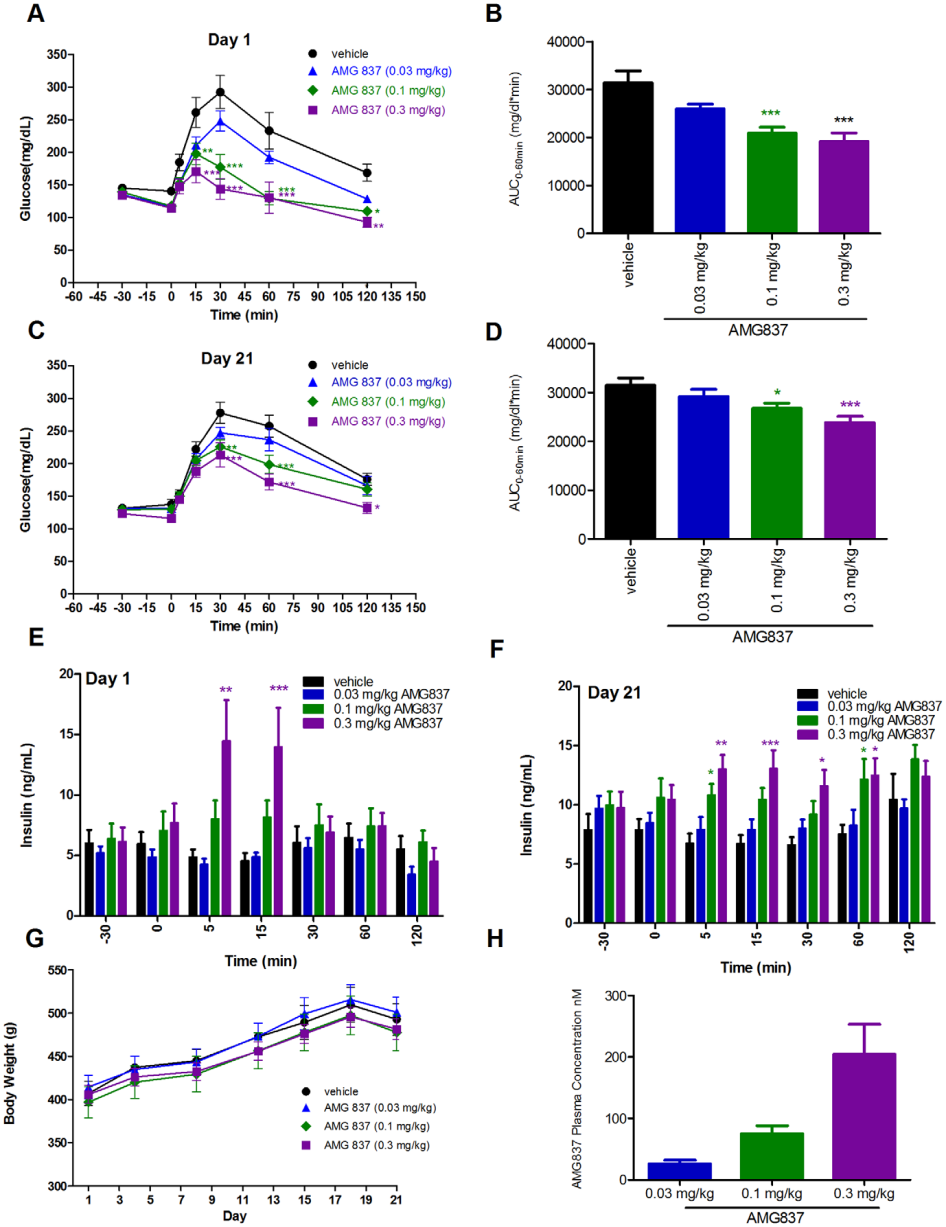
Efficacy of AMG 837 in Zucker fatty (*fa/fa*) rats following daily dosing for 21-days. 8-week old Zucker fatty rats were administered a single bolus of AMG 837 (at 0.03, 0.1 and 0.3 mg/kg, n = 6/group) by oral gavage 30-minutes prior to an intraperitoneal glucose challenge at t = 0 minutes. (A) Blood glucose during the IPGTT (black circle = vehicle, blue triangle = 0.03 mg/kg AMG 837, green diamond = 0.1 mg/kg AMG 837 and purple square = 0.3 mg/kg AMG 837). (B) Glucose AUC (from −30 to 120 minutes) during the IPGTT. (E) Plasma insulin levels during the IPGTT (black bar = vehicle, blue bar = 0.03 mg/kg AMG 837, green bar = 0.1 mg/kg AMG 837 and purple bar = 0.3 mg/kg AMG 837). Once daily dosing was continued for 21-days. On day 21, an IPGTT was performed in an identical manner to that on day 1. (C) Blood glucose (D) glucose AUC (from −30 to 120 minutes) and (F) plasma insulin levels were measured from the day 21 IPGTT. Figure legends are identical to those of the day 1 figures. (G) Body weights of the animals were followed through the course of the 21-day study; no difference in BW was observed between the groups. (H) Total plasma concentration of AMG 837 30-minutes following the final dose on day 21. Statistical significance compared to vehicle treated animals is denoted by * (p<0.5), ** (p<0.01) and *** (p<0.001) as determined by one-way or two-way ANOVA.

In order to understand the effect of AMG 837 following multiple doses, AMG 837 was dosed at 0.03, 0.1 and 0.3 mg/kg by oral gavage daily for 21-days. Thirty minutes following the first dose, an IPGTT was performed. AMG 837 improved glucose levels during the IPGTT (figure 5A) with a decrease in glucose AUC of 17%, 34% (p<0.001), and 39% (p<0.001) at 0.03, 0.1 and 0.3 mg/kg, respectively (figure 5B). This was associated with increased insulin secretion following glucose administration (figure 5E). Because a separation in the pharmacological response to glucose challenge could be observed below but not above 0.3 mg/kg (figure 4), this indicates that 0.3 mg/kg is approximately the maximal dose in this rat model.

Administration of AMG 837 was continued daily for 21-days in order to test the effects of AMG 837 following multiple doses. A second IPGTT was performed 30 minutes following the final dose on day 21 and AMG 837 lowered glucose levels following glucose challenge (figure 5C). Glucose AUC values during the GTT were decreased to 7%, 15% (p<0.05), and 25% (p<0.001) at 0.03, 0.1 and 0.3 mg/kg, respectively (figure 5D). Insulin levels prior to glucose challenge at day 21 were higher in all groups compared to those on day 1, likely indicative of progressive insulin resistance in these animals. In rodents treated with AMG 837, insulin levels increased in the mid- and high- dose groups post-glucose challenge (figure 5F). Body weights were not affected by AMG 837 treatment during the 21-day treatment (figure 5G). Taken together, these results indicate that the pharmacological activity of AMG 837 persisted even after 21-days. Total plasma concentrations of AMG 837 30-minutes following the final dose of 0.03 mg/kg, 0.1 mg/kg and 0.3 mg/kg AMG 837 were 26±6 nM, 75±13 nM and 204±49 nM, respectively (figure 5H).

## Discussion

The GPR40 (FFA1) receptor appears to be a viable and amenable target for small molecule development for use in treating type 2 diabetes. Several GPCRs expressed on the pancreatic β-cell, such as GPR119 and GPR40 have gained considerable interest as drug targets [26], [27]. Because of the success of treating diabetics with drugs that impact the GLP-1R pathway, it is envisioned that drugs targeting other β-cell GPCRs may offer a new treatment option. Interestingly, both GPR40 and GPR119 are also expressed on the enteroendocrine cells of the gastrointestinal tract and lead to stimulation of gut hormones such as GLP-1. Studies of the effect of AMG 837 on gut hormones are currently underway.

In addition to the expression on the β-cell and enteroendocrine cells, GPR40 expression has also been described in osteoclasts [28], pancreatic α-cells [29], taste buds [30], immune cells [2] and specific neurons in the brain [31]. AMG 837 may activate GPR40 receptors in these tissues, but since AMG 837 is a partial agonist, the pharmacological effect will be more sensitive to receptor expression levels than that for a full agonist. AMG 837 may activate GPR40 receptors in the brain. However, the exposure of AMG 837 in the CNS was not measured. The physicochemical properties of AMG 837 are generally consistent with blood-brain barrier penetration. The polar surface area of the compound is 46 Å^2^, which is within the preferred range of <70 Å^2^ for CNS penetrating drugs [32], [33]. The molecular weight ( = 438 Daltons) and ClogD_7.4_ ( = 3.7) of AMG 837 are also within the limits suggested for compounds that penetrate the CNS [34].

Several synthetic GPR40 agonists have been described in recent years, including GW9508, TAK-875, TUG-424 and others [9], [10], [13], [15]. Broadly speaking, these agonists, including AMG 837, share similar structural features: a carboxylic acid group or carboxylate bioisostere separated from a substituted aryl ring by two carbon atoms. While side-by-side pharmacological comparisons of these agonists have not been described, these GPR40 agonists, in general, potentiate GSIS and improve post-prandial glucose in various rodent models. Two agonists, TAK-875 and the molecule described here, AMG 837, have been disclosed as clinical candidates for the treatment of type 2 diabetes.

AMG 837 is a partial GPR40 agonist that potently activated GPR40 in cell-based assays and isolated islets. AMG 837 did not potentiate insulin secretion in islets from GPR40 knockout mice (figure 2B) and the improvement during an OGTT was lost in GPR40 null mice compared to that in wild type mice (Houze JB *et al*, in preparation). The activity of AMG 837 *in vitro* was right-shifted in the presence of albumin or serum. While plasma protein binding reduces the levels of free AMG 837, sub-µM plasma levels of total AMG 837 (figure 5H) in the Zucker fatty rat were sufficient to reduce glucose levels. This result indicates that at least the rat is quite sensitive to AMG 837.

One important aspect of any GPR40 agonist that may be used to treat a chronic condition such as type 2 diabetes is the potential for tachyphylaxis. Results from two experiments addressed this potential. First, a single dose of AMG 837 improved glucose levels following consecutive glucose challenges, indicating a lack of acute tachyphylaxis (figure 3D–F). Second, administration of AMG 837 for 21-days *qd* demonstrated continued efficacy in Zucker fatty rats (figure 5). While prolonged studies in the clinic will ultimately establish the utility of long-term dosing of GPR40 agonists, these preclinical data with AMG 837 are encouraging with a respect to a lack of tachyphylaxis. AMG 837 did not affect body weight (figure 5G), indicating that improvements in glucose excursion following 21-day dosing were independent of body weight. It should be noted that the improvements in glycemic parameters observed with 21-day treatment with AMG 837 run counter to the notion that prolonged agonism of GPR40 is linked to worsening glucose control [35].

In conclusion, we have discovered and characterized a novel GPR40 (FFA1) agonist, AMG 837. Further studies of AMG 837 will delineate the biological functions of GPR40 and whether the GPR40 pathway can be modulated to treat human disease.

## Materials and Methods

### Plasmids and cell lines

GPR40 was amplified from genomic DNA using standard PCR techniques and cloned into pcDNA3.1 (Invitrogen) or the retroviral vector pLPC (from Dr. David Mu). Constructs were verified by DNA sequencing. An A9 cell line stably transfected with pLPC-GPR40 (A9_GPR40) was created by retroviral infection of mouse A9 cells (ATCC catalog # CRL-1811) followed by selection on 2 µg/mL puromycin (Sigma-Aldrich) as described [36]. Chinese hamster ovary (CHO, ATCC catalog # CCL-61) cells were transiently transfected with pCDNA3.1-GPR40 using lipofectamine 2000 (Invitrogen).

### AMG 837

AMG 837 (*(S)*-3-(4-((4′-(trifluoromethyl)biphenyl-3-yl)methoxy)phenyl)hex-4-ynoic acid) was synthesized by the Amgen Chemistry Research and Discovery Department, South San Francisco, CA. Synthesis and characterization of AMG 837 were conducted as described (Houze, JB *et al*, in preparation and [37]). AMG 837 was >98% pure as judged by HPLC area integration with UV detection at a wavelength of 254 nM.

### GTPγS binding assay

A GTPγS binding assays using an anti-G_α_-protein scintillation proximity assay format was employed essentially as described [23]. Assays were performed in Corning 96-well plates (Corning catalog #3604). Cell membranes were prepared from an A9 cell line stably transfected with GPR40 (A9_GPR40). Cell membranes were mixed with various concentrations of AMG 837, 0.1 µM GDP, 400 pM [^35^S]-GTPγ (Perkin-Elmer) in binding buffer (consisting of 20 mM Hepes pH 7.4, 100 mM NaCl and 5 mM MgCl_2_) in a volume of 200 µl/well. Plates were incubated for 60 minutes at room temperature. Next, 20 µl of 3% NP-40 were added to each well and the plates were further incubated for 30 minutes. This was followed by the addition of 20 µl of anti-G_q_ antibody (anti G_α q/11_ antibody, Santa Cruz Biotechnologies cat # SC-392, 1∶400 dilution) and the plates were incubated for an additional 60 min. Finally, 50 µl of anti-rabbit-SPA beads (Amersham # RPNQ 0016) were added to each well and the plates were incubated for 3 hrs. Antibody captured [^35^S]-GTPγ was measured using a Microbeta (Wallac).

### Inositol phosphate accumulation assays

A9_GPR40 cells were plated in 96-well plates containing 20,000 cells/well in DMEM containing 10% FBS. After the cells attached to the well surface, the media was replaced with inositol free DMEM containing 10% dialyzed FBS and 1µCi, mL ^3^H-myo-inositol and incubated for 16 hours. Compounds were diluted in HBSS/10 mM LiCl, pH7.4 in 0.01% HSA and added directly to cells. Following 1 hour incubation at 37°C, the media was replaced with 100 µl of 20 mM formic acid to quench the reaction. 50 µL of the extract was then added to 100 µL of SPA beads, incubated overnight, and measured on a TopCount the following day.

### Aequorin assay

CHO cells were plated in 15 cm plates containing 8×10^6^ cells/plate in DMEM/F12 containing 10% FBS. The following day, cells were transfected with 5 µg of GPR40 expression plasmid and 5 µg of aequorin expression plasmid (Euroscreen) complexed with 30 µL of Lipofectamine 2000. In plasmid titration experiments, the amount of GPR40 expression plasmid was reduced, but the total amount of DNA transfected was kept constant by adding in empty vector DNA. Sixteen to twenty-four hours post-transfection, cells were washed with PBS and detached from the plate with 2 mL trypsin (0.25% in HBSS). 28 mL of HBSS containing a desired amount of HSA (0.01% or 0.625% w/v, Sigma-Aldrich) or human serum (100% v/v, Sigma-Aldrich) was added to the detached cells and coelenterazine was added to final concentration of 1 µg/mL. Cells were allowed to incubate in coelenterazine containing buffer for 2 hours prior to assay. AMG 837 and DHA (Sigma-Aldrich) stock solutions were prepared in DMSO and then diluted in HBSS buffer containing the % HSA identical to that in which the cells were incubated in. Compounds were allowed to complex with HSA for 1 hr at 37°C. Aequorin activity was measured using a microlumat.

### Isolation of Mouse Pancreatic Islets

Islets were prepared from mouse pancreas following injection of collagenase into the common bile duct followed by purification on a histopaque gradient. Animals were euthanized by CO_2_ inhalation and the abdominal cavity was opened. The common bile duct was clamped just proximal to the duodenum and the pancreas was perfused with 3–5 mL of ice cold collagenase (0.67 mg collagenase/mL in HBSS containing 25 mM Hepes pH 7.4 and 1% penicillin/streptomycin). The inflated pancreas was excised and collagenase digestion was allowed to proceed for 20 minutes in a 37°C water bath. The digestion was quenched by addition of quenching buffer (10% FBS in HBSS containing 25 mM Hepes pH 7.4 and 1% penicillin/streptomycin). Islets were washed twice with quenching buffer following centrifugation at 300×g for 2 minutes. Islets were pelleted and resuspended in 10 mL of Histopaque 1119 (Sigma-Aldrich). Next, 10 mL of Histopaque 1077 was layered on top of the Histopaque 1119 layer, and 10 mL of quenching buffer was carefully layered on the very top. The tube was centrifuged at 1000×g for 30 minutes and the islets were isolated with a pipet and washed in culture media (RPMI modified, 10% FBS, 25 mM Hepes, 1% penicillin/streptomycin, pH 7.4, 37°C). Islets were allowed to culture for 48 hours in a cell incubator and were then handpicked under a dissection microscope and transferred to a 96-well transwell plate (Corning). Insulin secretion assays were performed in KRBH, pH7.4 (consisting of 129 mM NaCl, 4.8 mM KCl, 1.2 mM KH_2_PO_4_, 1.2 mM MgSO_4_, 10 mM HEPES, 2.5 mM CaCl_2_, 25 mM NaHCO_3_) and the insulin secreted into the supernatant was measured using an insulin ELISA (Alpco).

### Animals

All procedures on animals were approved by the Amgen San Francisco Institutional Animal Care and Use Committee (approved protocol #11-04). Eight week old male Sprague-Dawley (SD) rats (Harlan, Indianapolis, Indiana) and 8-week old male Zucker fatty rats (*fa/fa*, Harlan, Haslett, MI; Barrier 206) were used in studies. Animals were maintained on a standard chow diet (Harlan Tecklad 2918, Madison, Wisconsin) and acclimated to the Amgen San Francisco facility (an AAALAC accredited facility) for a minimum of 7-days before treatment. Animals were housed in Tecniplast cages on a ventilated rack. The animals were housed under a 12-h light, 12-h dark cycle (lights-on 0600 h and lights-off 1800 h) and were allowed *ad libidum* access to regular chow and water.

### *In vivo* procedures

AMG 837 was formulated for oral dosing using 1% methylcellulose (CMC), 1% Tween 80 (Sigma-Aldrich, St. Louis, MO). For evaluation of AMG 837 following a single dose in rats, animals were fasted overnight and then randomized into dose groups based on their body weights. Thirty minutes after oral administration of their respective treatments, the animals received a 1 g/kg glucose challenge dose by intraperitoneal injection. Blood samples were collected at 0, 5, 15, 30, 60, and 120 minutes via tail vein after the glucose challenge. Glucose levels were monitored with a Glucometer (Elite XL). Plasma insulin was measured using a rat insulin ELISA kit (ALPCO Diagnostics, Windham, NH). For evaluation of AMG 837 in Zucker fatty rats, animals were randomized based on body weight and received either vehicle, 0.03 mg/kg, 0.1 mg/kg, or 0.3 mg/kg AMG 837 once daily for 21 days by oral gavage. Treatments were administered between 0900 and 1000 h during the light cycle. On days 1 and 21, an intraperitoneal glucose tolerance test (IPGTT) was performed as described above.

### Bioanalytical Analysis

AMG 837 plasma concentrations were measured using a sensitive and selective LC/MS/MS method. Briefly, following the addition of internal standard, samples were extracted using protein precipitation. The resulting supernatants were dried, reconstituted and injected into a a triple quadruple LC/MS/MS instrument (API 3000, AB Sciex, Foster City, CA) for detection. Concentrations of AMG 837 in plasma samples were calculated using a calibration curve with a lower limit of quantitation of 1 ng/mL.

### Statistics

Data was expressed as mean ± SEM. One- or two-way ANOVA (GraphPad Prism) was used to assess statistical significance between control and treatments.
